# Engineering an Artificial Metalloenzyme for Efficient Photo‐Enzymatic CO_2_ Conversion

**DOI:** 10.1002/advs.78027

**Published:** 2026-09-27

**Authors:** Mengxue Kang, Yan Chu, Zhichun Xu, Jiao Feng, Ganlu Li, Kequan Chen, Hui Li

**Affiliations:** ^1^ College of Biotechnology and Pharmaceutical Engineering State Key Laboratory of Materials–Oriented Chemical Engineering Nanjing Tech University Nanjing China

**Keywords:** CO_2_ conversion, dual catalytic centers, light‐driven artificial metalloenzyme, NADH regeneration

## Abstract

Integrated photo‐enzymatic catalysis combines the merits of both photocatalytic and enzymatic systems; however, the spatial separation of photocatalysts and enzymes in conventional one‐pot setups often limits overall efficiency due to sluggish electron transfer. To address this, we constructed a light‐driven artificial metalloenzyme (Rh‐L6‐FDH) featuring dual catalytic centers by covalently conjugating formate dehydrogenase (FDH) with a tailored rhodium complex (Rh‐L6), thereby facilitating synergistic NADH regeneration and CO_2_ conversion. Screening nine synthesized Rh complexes with structurally diverse ligands revealed that Rh‐L6 exhibited the highest efficiency for light‐driven NADH regeneration. Consequently, the resulting Rh‐L6‐FDH conjugate yielded formate with a 2.31‐fold increase compared to the physical mixture of free FDH and Rh‐L6. The enhancement is mechanistically linked to the combined effects of a narrowed bandgap, diminished activation energy, and the proximity between catalytic centers, which synergistically promote charge separation and CO_2_ activation. To overcome the inherent instability of the conjugate, Rh‐L6‐FDH was immobilized within a ZIF‐67 framework. This strategy markedly improved catalytic robustness, boosting formate conversion to 98.4%—a 16.68‐fold improvement over the free component system. This work demonstrates the potential of integrating molecular metal complexes with enzymes into a unified architecture, offering a promising pathway for sustainable cofactor regeneration and efficient carbon sequestration.

## Graphical abstract

We built a light‐driven artificial metalloenzyme by covalently conjugating formate dehydrogenase (FDH) with a tailored rhodium complex (Rh‐L6) to overcome spatial‐separation‐limited electron transfer. This integration boosts NADH regeneration and CO_2_ conversion, giving 2.31‐fold higher formate yield than physical mixtures, due to a narrowed bandgap and reduced activation energy.

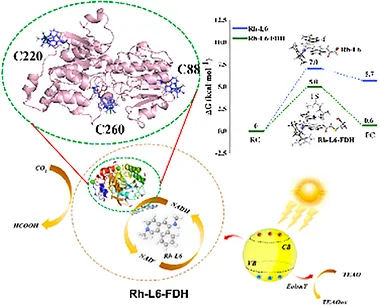

## 1 Introduction

Light‐driven CO_2_ reduction harnesses photo‐generated electrons to regenerate reducing equivalents, facilitating the conversion of CO_2_ into value‐added chemicals [1]. Unlike conventional photochemical methods—which often struggle to mediate complex reduction pathways and rely on energy‐intensive, high‐temperature calcined photocatalysts—biophotocatalysis offers a greener alternative with operating under mild conditions, lowers costs, and expanding the range of accessible products [1, 2, 3]. Inspired by natural photosynthesis, researchers have integrated enzymes with photocatalysts to construct photoenzymatic systems [2, 3, 4, 5]. In these hybrid designs, photocatalysts either supply additional reducing power to CO_2_‐reducing enzymes or directly channel photo‐generated electrons for NADH regeneration, thereby driving redox reactions and enabling efficient CO_2_ conversion [5]. Nevertheless, the diffusion distance of electrons or intermediate substances remains a critical bottleneck, limiting overall chemical yields.

Rhodium complexes have emerged as key mediators for selective NADH regeneration, enhancing system sustainability when combined with cocatalysts. This synergistic integration, based on complementary functions, shortens electron transfer pathways and improves photocatalytic performance [6]. For instance, Bhat et al. incorporated rhodium into BaTiO_3_ photocatalysts, effectively narrowing the bandgap and significantly boosting photocatalytic activity, achieving up to 96% degradation of methylene blue dye in a short period [7]. Song et al. reported that rhodium phosphide as a cocatalyst on Cd‐based photocatalysts exhibits strong electron‐accepting ability, accelerating the separation of photoinduced electron–hole pairs and thereby enhancing H_2_ evolution performance [8]. Similarly, Zhang et al. immobilized rhodium complexes on porous carbon electrodes for efficient NADH regeneration [9]. Overall, rhodium complexes are widely utilized in photocatalytic systems to overcome the limited activity of inert photocatalysts. They enhance photoactivity by facilitating photogenerated electron transfer, while their ability to mediate proton transfer promotes the reduction of NAD^+^to NADH.

Through millions of years of evolution, enzymes have acquired the ability to catalyze a diverse array of complex biochemical reactions. While proteins consisting only of amino acids exhibit a limited catalytic scope, approximately one‐third of natural enzymes incorporate cofactors or metal ions within their active sites to facilitate more sophisticated chemical transformations [10, 11, 12]. Inspired by this, researchers have begun integrating synthetic metal cofactors into protein or enzyme scaffolds to construct artificial metalloenzymes (ArMs), thereby expanding catalytic activity to non‐natural reactions [13, 14]. Common strategies for constructing ArMs include metal coordination with amino acid residues, non‐covalent binding of metal complexes, and covalent attachment via functionalized metal complexes [15, 16, 17]. For example, Okamoto et al. developed a light‐driven ArM by incorporating the DNA photo‐opening metallocomplex [Ru(bpy)_2_dppz]^2+^ into the *apo*‐form of riboflavin‐binding protein, enabling switchable control between photoredox and energy transfer pathways through light regulation [18]. The ligand architecture and linker length of metal complexes are key determinants of catalytic efficiency. Through rational design, ArMs hold great promise in addressing current challenges in photo‐enzymatic catalysis, such as limited overall efficiency and the kinetic mismatch between enzymatic and photocatalytic steps.

Formate dehydrogenase (FDH) from *Ceriporiopsis subvermispora* serves as a key biocatalyst for CO_2_ conversion, yet its practical application is often limited by the requirement for costly NADH as an electron donor. In this work, through rational ligand and linker design, a series of rhodium complexes with varied structures were synthesized and covalently conjugated to FDH via specific amino acid residues, producing artificial metalloenzymes (ArMs) capable of efficient NADH regeneration and CO_2_ reduction. Among them, Rh‐L6‐FDH serves as a bifunctional ArM that integrates enzymatic CO_2_ conversion with light‐driven NADH regeneration in a single construct. Furthermore, encapsulation of Rh‐L6‐FDH within a ZIF‐67 framework enhanced operational stability by shielding the biocatalyst from detrimental microenvironmental fluctuations and allowed its reuse over multiple catalytic cycles.

## 2 Results and Discussion

### 2.1 Synthesis and Structure of Bifunctional Rh‐L6‐FDH

**FIGURE 1:**
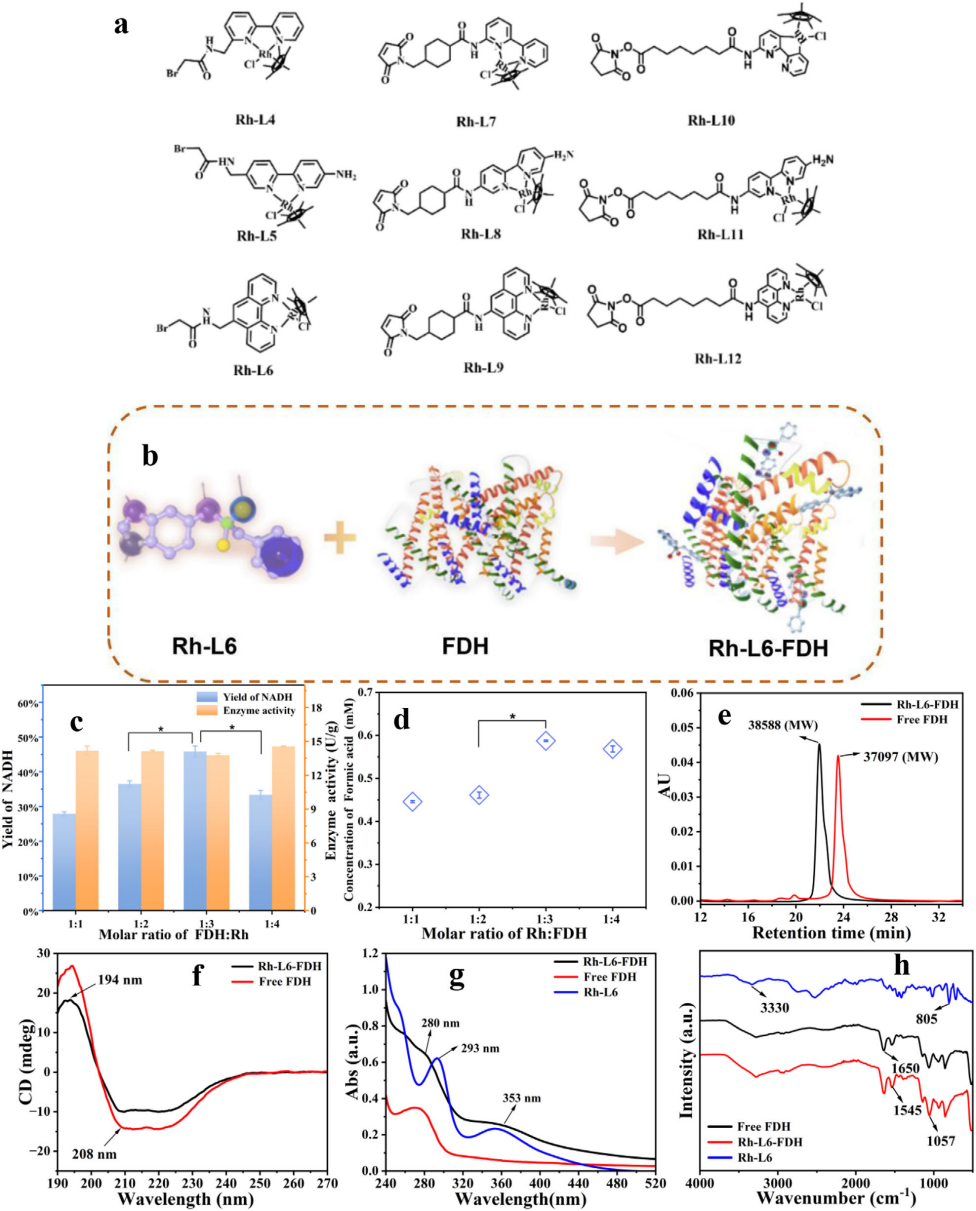
Synthesis and structure of bifunctional Rh‐L6‐FDH. (a) Rh complexes with different ligands. (b) Schematic diagram of the artificial metalloenzyme (ArM) **Rh‐L6‐FDH**. (c) Effect of molar ratios of FDH: Rh‐L6 on enzyme activity and NADH regeneration. Error bars: mean ± SD (*n* = 3). Statistical significance: *p < 0.05 (t‐test). (d) Effect of molar ratios of FDH: Rh‐L6 on formate production. Error bars: mean ± SD (*n* = 3). Statistical significance: *p < 0.05 (t‐test). (e) Gel permeation chromatography (GPC) profiles of **Rh‐L6‐FDH** and free FDH, (f) Circular dichroism (CD) spectra of **Rh‐L6‐FDH** and free FDH, (g) Ultraviolet‐visible (UV–vis) spectra of **Rh‐L6‐FDH**, free FDH and Rh‐L6, (h) Fourier transform infrared spectroscopy (FTIR) spectra of **Rh‐L6‐FDH**, free FDH and Rh‐L6.

The conversion of CO_2_ by FDH requires the coenzyme NADH to supply H^+^. However, NADH is costly and prone to oxidation in solution, making its direct use inefficient. To reduce costs, NAD^+^ is typically recycled to NADH prior to catalysis [19]. Rhodium complexes serve as key mediators for the selective regeneration of NAD^+^ [20]. Building on this strategy, rhodium complexes were coupled with varying ligand molecules and linker lengths (Figure 1a), which enhance NADH regeneration rates, with FDH to successfully construct a rhodium‐based dual catalytic centers ArM using FDH as the protein scaffold. This bifunctional ArM improves NADH recycling during catalysis and significantly enhances substrate conversion efficiency.

Nine rhodium complexes were designed and conjugated to FDH via distinct binding modes (See Supplementary Methods and Figures S1–S9). The enzymatic activity of FDH and the NADH regeneration capacity were influenced by variations in ligand architecture and conjugation sites. Most complexes maintained a specific enzyme activity of approximately 15 U/g, except for Rh‐L10‐FDH, which showed a slight decrease to about 12 U/g, likely due to perturbation of the FDH active site upon conjugation. All nine ArMs achieved NADH conversion efficiencies around 20%, among which Rh‐L6‐FDH exhibited the highest value of 27.8%—a 2.31‐fold increase over the free FDH + Rh system (Figure S10a). Complexes Rh‐L4−L6, conjugated to FDH through bromoacetamide groups with shorter ligand spacers, demonstrated reduced electron transfer distances and improved catalytic efficiency. Rhodium complexes bearing phenanthroline ligands (e.g., Rh‐L6) facilitate electron transfer more effectively than those with bipyridine analogues [21]. Enhanced NADH regeneration promoted CO_2_ conversion, with Rh‐L6‐FDH yielding the highest concentration—twice that of the free FDH + Rh benchmark (Figure S10b).

The Rh‐L6‐FDH was synthesized by covalently linking the bromoacetamide group of Rh‐L6 to the thiol groups of Cys residues on FDH (Figure 1c) [22]. Given that FDH contains four Cys residues, multiple binding configurations were possible. The molar ratio of FDH to Rh‐L6 did not affect the catalytic activity of FDH, which remained stable at 15 U/g (Figure 1c). At a 1:3 molar ratio of FDH: Rh‐L6, the higher concentration of the Rh‐L6 yielded the highest NADH regeneration rate. The formic acid synthesis process achieved an optimal balance between the NADH consumption rate and the rhodium complex‐promoted NADH regeneration rate (0.031 mM∙min^−1^). Under these conditions, formate production reached a maximum. The proximity between the metal catalytic center and the enzymatic catalytic center enables regenerated NADH to quickly access the enzymatic catalytic center, thereby facilitating formate synthesis. However, excessive amounts of the rhodium complex posed a risk of damaging the enzyme's active site, as indicated by the sharp decline in formic acid yield (Figure 1d) observed at a 1:4 molar ratio.

Sodium dodecyl sulfate polyacrylamide gel electrophoresis (SDS‐PAGE) was employed to verify the molecular weights of free FDH and Rh‐L6‐FDH (Figure S10c). Two distinct bands were observed at about 40 kDa, with the Rh‐L6‐FDH band appearing slightly higher than that of free FDH, confirming successful conjugation [23]. SDS can disrupt protein structures and release non‐covalently bound substances. The distinct band differences indicate that the rhodium complexes have formed a covalent bond with FDH. Gel permeation chromatography analysis (Figure 1e) revealed that Rh‐L6‐FDH eluted earlier than free FDH, indicating a larger molecular weight. The observed difference corresponded to about 2.5 times the mass of a single Rh‐L6 unit with conjugation efficiency of 83%, suggesting that 2–3 rhodium complexes were bound per enzyme molecule. Electrospray ionization‐mass spectrometry (ESI‐MS, Figure S10d) further confirmed these results, with free FDH measured at 36 759.12 Da and Rh‐L6‐FDH at 38 483.32 Da, consistent with the attachment of three Rh‐L6 units [24]. The mass spectrum showed only two principal peaks, and no intermediates linking one or two Rh‐L6 moieties were detected. Furthermore, no evidence of nonspecific binding or oligomeric byproducts was observed. This clearly demonstrates that Rh‐L6‐FDH possesses a unique and unambiguous attachment pattern, with high homogeneity, allowing the product to be used directly for activity measurements without further purification.

The tertiary structure of free FDH and Rh‐L6‐FDH was analyzed using circular dichroism (CD) spectroscopy (Figure 1h). The Rh‐L6‐FDH complex exhibited a more pronounced positive peak at 194 nm and a stronger negative peak at 208 nm, indicating an increase in *α*‐helices content. These findings suggest that the interaction between the bromoacetamide group of the rhodium complexs and FDH induced changes in the enzyme's secondary structure. The CD spectra were calculated to obtain the specific protein structure data (Table S1), from which it can be seen that the *β*‐sheet is the main conformation of the FDH protein molecule, and the number of this structure decreases after the coupling of the rhodium complex. Combined with the CD spectra, the main change in the secondary conformation of FDH after the synthesis of ArM is a 4% increase in the number of *α*‐helices and a relative decrease in the number of *β*‐sheets.

The compositions of FDH, Rh‐L6, and Rh‐L6‐FDH were analyzed via ultraviolet‐visible (UV–vis) spectroscopy (Figure 1g). The Rh‐L6 presented two characteristic absorption peaks at 293 and 353 nm, the former representing the bromoacetamide group of the rhodium complex, the latter representing the phenanthroline group [25]. The FDH at 280 nm has one characteristic absorption peak representing the protein characteristic fraction, whereas Rh‐L6‐FDH obviously has maximum absorption peaks at about 280 and 353 nm compared with those of FDH, indicating that the Rh‐L6 has been successfully coupled and that the ArM has been successfully constructed. Fourier transform infrared spectroscopy (FTIR) (Figure 1h) showed the peaks of Rh‐L6‐FDH at 1650 and 1545 cm^−1^ belonged to the protein‐specific amide I and amide II bands, which originated from the amide bonds of proteins. The peak at 805 cm^−1^ represented the C─H stretching vibration of the benzene ring on the rhodium complex, and the peak at 3330 cm^−1^ represented the N─H stretching vibration. The peak at 1057 cm^−1^ is characteristic of the sulfide‐ether bond, which is more intense than that of FDH, suggesting that the sulfhydryl group on Cys covalently combines with bromoacetamide to generate a new sulfide‐ether bond.

**FIGURE 2:**
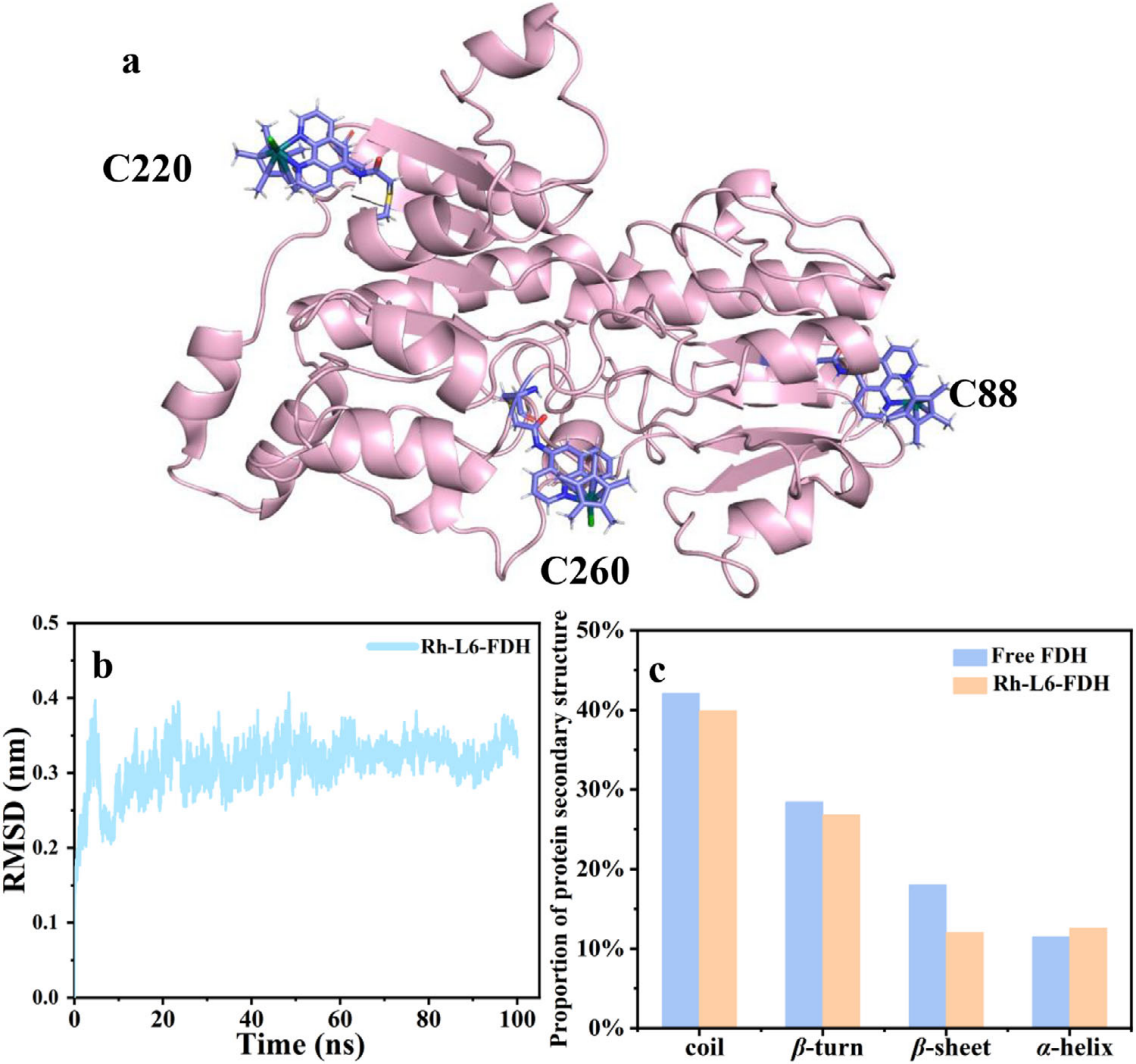
Binding characteristics of bifunctional Rh‐L6‐FDH. (a) Model construction results of the covalent combination of Rh‐L6 and FDH, (b) Changes in the RMSD of **Rh‐L6‐FDH** over time, (c) Simulation of the content of the protein secondary structure after the combination of FDH and **Rh‐L6‐FDH**.

The FDH used in this study contains four cysteine residues. Based on the optimal conformation, FDH should couple with Rh‐L6 at a molar ratio of 1:3. The Rh‐L6‐FDH model was constructed according to the binding energy of the rhodium complex to three of the four Cys sites—C88, C220, and C260 (Figure 2a). Protein stability of the ArM was evaluated using root‐mean‐square deviation (RMSD). The overall RMSD of the protein converged around 0.3 nm, with fluctuations within 0.1 nm (Figure 2b). Significant RMSD variations during the first 10 ns of simulation indicate that the introduction of Rh‐L6 induced structural rearrangements in the early stage, after which the overall structure stabilized. Radius of gyration (Rg) analysis (Figure S11a) showed that the protein's overall Rg remained stable at approximately 2.20 nm. Consistent with the RMSD profile, some initial conformational fluctuations were observed; however, these did not lead to major structural changes. This suggests that incorporation of the rhodium complex did not markedly perturb the native FDH fold or compromise the overall protein integrity. Root‐mean‐square fluctuation (RMSF) values across the protein were generally low (Figure S11b). The three attachment sites for the non‐canonical residues exhibited RMSF values below 0.1 nm, indicating local structural stabilization upon residue incorporation. Analysis of intra‐protein hydrogen bonds revealed that during the initial simulation phase (first 10 ns), structural adjustments led to a decrease in hydrogen‐bond count from about 300 to 260. As the structure gradually stabilized, the final number of hydrogen bonds recovered to 282 (Figure S11c). Changes in the number of hydrogen bonds indicate alterations in the secondary structure composition of the FDH molecule. The solvent accessible surface area (SASA), which describes the biomolecular surface area exposed to solvent, was also examined. Results indicate that following the introduction of non‐canonical residues, the SASA of the protein decreased, suggesting a reduction in overall polarity (Figure S11d). The nonpolar characteristics of Rh‐L6 reduce the polarity of Rh‐L6‐FDH.

Analysis of the residue composition over the simulation time reveals that no large‐scale transitions occurred in the overall secondary structure of the protein (Figure S11e). However, upon closer inspection, it can be observed that among the three sites connected to the non‐canonical residue: in the vicinity of C88, the secondary structure composition remains relatively stable after connection, though some interconversion between Turn and *α*‐helix is present; near C220, more pronounced mutual transitions among α‐helix, Turn, and Bend are evident; around C260, certain interconversions between 3‐helix, Turn, and Bend are detected. Additionally, in the region near residue 150, relatively distinct transitions between α‐helix and Turn are also observed. The secondary structure content after molecular simulation was calculated using the definition of secondary structure of proteins program (Figure 2c). The incorporation of Rh‐L6 alters the weak interaction network of the protein molecule, which induced subtle alterations in the enzyme's secondary structure, characterized by an increase in *α*‐helices and a corresponding decrease in *β*‐turns, *β*‐sheets, and random coils. The hydrophobic nature of Rh‐L6 likely strengthens intramolecular hydrophobic interactions within FDH, thereby shielding helical hydrogen bonds from water‐mediated disruption and enhancing overall stability [26]. These simulation results were consistent with the conclusions drawn from the CD analysis described earlier, confirming that the conformation of FDH, as the protein scaffold, underwent slight modifications during the synthesis of the ArM.

Here, a novel rhodium‐based bifunctional ArM was first developed that enables efficient NADH regeneration during light‐driven CO_2_ reduction. This integrated system significantly mitigates the dependence on expensive NADH cofactors while substantially boosting formic acid production. In these rhodium complexes, the ligand framework critically modulates both electronic and steric properties. For instance, the phenanthroline ligand in Rh‐L6 raises the *d*‐orbital splitting energy at the rhodium center, enabling visible‐light absorption and promoting metal‐to‐ligand charge transfer (MLCT). Electron‐donating substituents (─NH_2_) further increase electron density at the metal, facilitating reduction steps and inducing a red shift in the MLCT band, indicative of lower‑energy charge transfer. Moreover, the bipyridine ligand provides substantial steric shielding, tailoring the microenvironment around the metal center. This geometry mitigates nonspecific interactions with solvents or external quenchers, thereby fine‐tuning electron transfer kinetics, improving reaction selectivity, and reinforcing catalytic stability. Additionally, the ligand facilitates the formation of a hydrophobic pocket that enhances substrate accessibility and stabilizes transition states, leading to synergistic gains in both operational stability and electron transfer efficiency.

### 2.2 Optical Properties of Bifunctional Rh‐L6‐FDH

**FIGURE 3:**
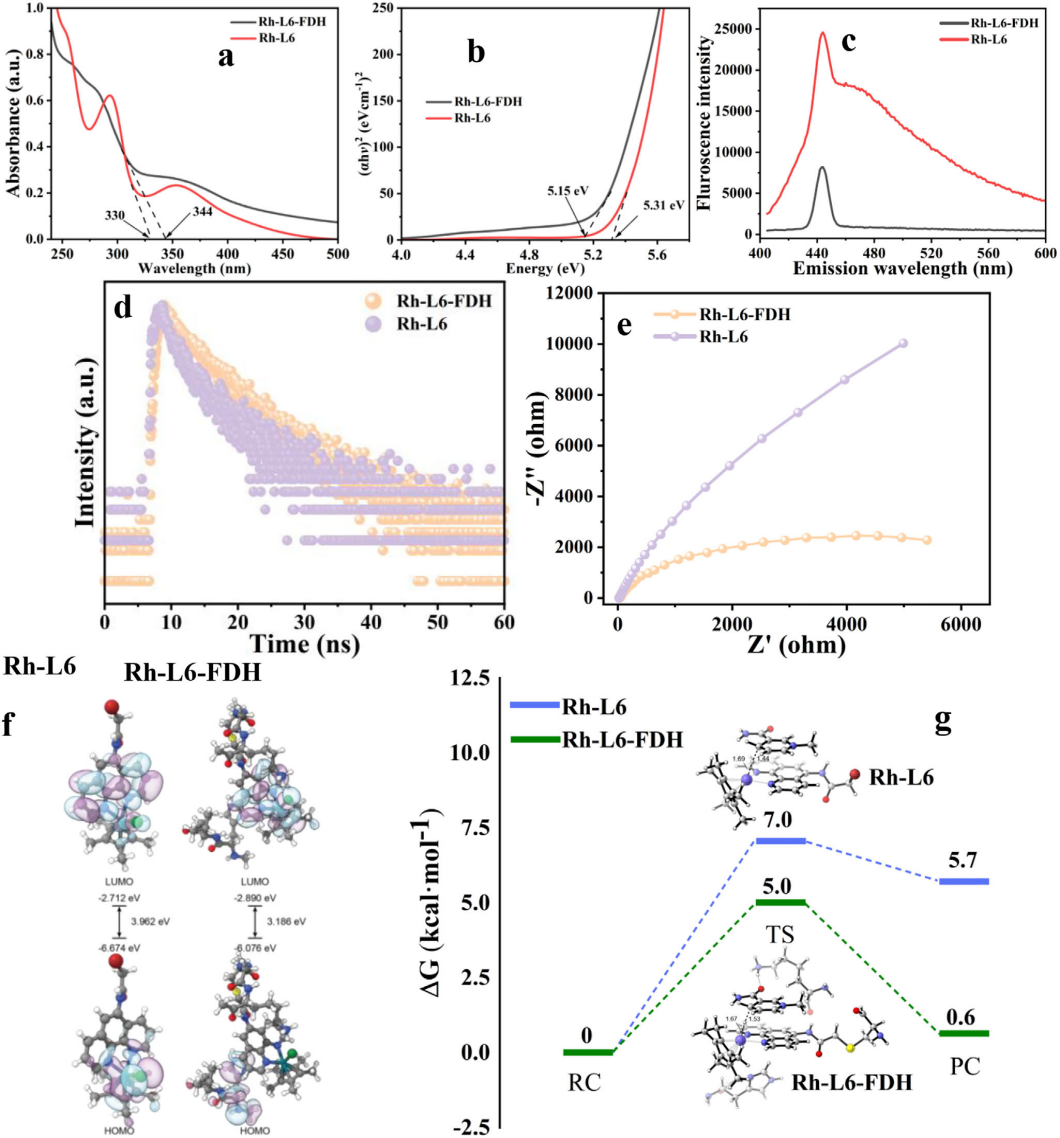
Optical properties of bifunctional Rh‐L6‐FDH. (a) The UV‐visible absorption spectrum of the artificial metalloenzyme **Rh‐L6‐FDH** and **Rh‐L6**. (b) The Tauc plot diagram of the artificial metalloenzyme **Rh‐L6‐FDH** and **Rh‐L6**. (c) Photoluminescence profile of **Rh‐L6‐FDH** and **Rh‐L6**. (d) Time‐resolved photoluminescence decay profile of **Rh‐L6‐FDH** and **Rh‐L6**. (e) Electrochemical impedance spectrum of **Rh‐L6‐FDH** and **Rh‐L6**. (f) Plots of the HOMO (Highest Occupied Molecular Orbital) and LUMO (Lowest Unoccupied Molecular Orbital) for the **Rh‐L6** and **Rh‐L6‐FDH** with the corresponding HOMO‐LUMO energy gap value indicated. (g) Reaction free energy profiles and transition state (TS) structures of the **Rh‐L6** and **Rh‐L6‐FDH**.

The absorption fringes and bandgaps of FDH and Rh‐L6‐FDH by UV–vis spectroscopy showed absorption edges near 309 and 344 nm, respectively, and Rh‐L6‐FDH clearly redshifted, indicating that Rh‐L6‐FDH has a stronger photoabsorption capacity than FDH and better optoelectronic properties, including a faster charge transfer rate, a lower charge charging compounding rate, and a narrower bandgap (Figure 3a). Based on the Tauc plot (Figure 3b), the optical bandgaps of FDH + Rh and Rh‐L6‐FDH were determined to be 5.51 and 5.21 eV, respectively. The smaller optical bandgap of Rh‐L6‐FDH indicates an expanded range of light‐absorbing wavelengths and an enhanced capacity to excite more charge carriers at room temperature, both of which are advantageous for photocatalytic applications [27].

The steady‐state luminescence emission spectra of Rh‐L6 and Rh‐L6‐FDH were utilized to investigate the photogenerated electron‐hole separation ability (Figure 3c). Under the same excitation at a wavelength of 375 nm, a strong fluorescence emission peak exists at 442 nm for Rh‐L6, and when the Rh‐L6 is coupled to free FDH, the fluorescence intensity is significantly reduced, indicating that electron transfer becomes faster during the catalytic process, which inhibits radiative complexation of the electron‐hole [28]. In addition, compared with Rh‐L6, the maximum fluorescence emission wavelength of Rh‐L6‐FDH at 445 nm exhibited a slight red shift, further indicating a higher degree of conjugation in Rh‐L6‐FDH; this observation was consistent with the UV–vis absorption spectrum. The influence of photogenerated carrier transport on the photocatalytic process was investigated using time‐resolved photoluminescence (Figure 3d). The fluorescence lifetimes of Rh‐L6 and Rh‐L6‐FDH were measured to be 1.59 and 1.54 ns, respectively. The reduction in fluorescence lifetime contributed to enhanced charge separation efficiency, thereby improving the photocatalytic performance of the ArM [29]. Electrochemical impedance spectrum results further demonstrated that the semicircle diameter of Rh‐L6‐FDH was smaller than that of Rh‐L6, indicating higher instantaneous carrier mobility and a more efficient suppression of electron‐hole radiative recombination (Figure 3e) [30].

Computational results indicate that Rh‐L6 and Rh‐L6‐FDH exhibit similar Lowest Unoccupied Molecular Orbital (LUMO) characteristics (Figure 3f). In all cases, the electron density is primarily delocalized over the phenanthroline ligand and the Rh center, suggesting that the lowest unoccupied molecular orbital is relatively insensitive to ligand modification and environmental factors. In contrast, significant differences are observed in the composition and energy levels of the Highest Occupied Molecular Orbital (HOMO) across these systems, which serves as the primary driver for variations in the HOMO‐LUMO gap. In Rh‐L6, the contribution from phenanthroline is significantly diminished, with electron density becoming more localized on the Rh center, the Cp* ligand, and the Cl ligand. This localization notably stabilizes the HOMO energy to −6.674 eV, resulting in a widened HOMO‐LUMO gap of 3.962 eV. In Rh‐L6‐FDH, upon integration into the protein environment, the electron‐rich side chain of amino acid residue contributes the highest occupied orbital, likely due to potential π−π stacking interactions between the amino acid (e.g., histidine) and the phenanthroline ligand. Consequently, the HOMO shifts entirely to the amino acid residue rather than the metal center. This elevation of the HOMO energy (−6.076 eV), introduced by the protein environment, significantly narrows the HOMO‐LUMO gap to 3.186 eV, which observations suggest a significant improvement in photocatalytic performance of Rh‐L6‐FDH.

To elucidate the impact of different structural models on the catalytic reduction of NAD^+^ to NADH, the free energy profiles were computed for the hydride transfer step from [(Cp∗)Rh(bpy)‐H]^+^ to NAD^+^. In these calculations, the cofactor models were truncated to the nicotinamide moiety to simplify the calculations (Figure 3g). The Rh‐L6‐FDH reveals a lower activation free energy (ΔG^‡^) of 5.0 kcal/mol. This reduction might originates from the stabilizing effects of the surrounding protein environment characterized in Rh‐L6‐FDH.

### 2.3 Synthesis and Catalytic Properties of Bifunctional Rh‐L6‐FDH@ZIF‐67

The bifunctional Rh‐L6‐FDH, though efficient in catalysis, tends to lose activity under harsh reaction conditions, leading to reduced formic acid yield and poor reusability. Zeolitic imidazolate frameworks (ZIFs) are known to enhance the structural stability of enzymes and help maintain their catalytic activity [31, 32, 33]. To address this, Rh‐L6‐FDH was immobilized within a ZIF‐67 framework via a one‐pot synthesis (Figure S12).

Scanning electron microscopy and transmission electron microscopy (TEM) were used to examine the morphological changes before and after immobilization (Figure S13a–d). Pristine ZIF‐67 exhibited irregular nanoflake structures, whereas after the incorporation of Rh‐L6‐FDH, the material transformed into compact, clustered microspheres with reduced particle size, confirming successful encapsulation of the enzyme within ZIF‐67 [34]. TEM‐based energy dispersive spectroscopy (EDS) mapping (Figure S13e,f) revealed a uniform distribution of nitrogen in both ZIF‐67 and Rh‐L6‐FDH@ZIF‐67, indicating successful ZIF‐67 synthesis. After immobilization, the content of O, N, and Rh increased by 8.21%, 2.13%, and 5.56%, respectively. These increases suggest the successful encapsulation of the enzyme and confirm the incorporation of the rhodium complex in Rh‐L6‐FDH@ZIF‐67 [35].

X‐ray photoelectron spectroscopy (XPS) further confirmed the presence of C, N, O, P, Co, and Rh on the surface (Figure S14). A peak at 399.52 eV corresponds to the N─Rh bond, while the intensified and right‐shifted peak at 530.95 eV is assigned to C═O. The P2p peak at 133.2 eV also showed a notable increase, and the Rh 3d region displayed characteristic doublet peaks at 305.09 and 311.45 eV. The elevated levels of N, O, and Rh, along with the presence of the N─Rh bond, verify the successful incorporation of Rh‐L6‐FDH into ZIF‐67 without structural disruption [36].

The structures and functional groups of ZIF‐67, Rh‐L6‐FDH, and the composite Rh‐L6‐FDH@ZIF‐67 were characterized by FTIR (Figure S15a). The spectra of Rh‐L6‐FDH and Rh‐L6‐FDH@ZIF‐67 display characteristic peaks at 1021 cm^−1^ (C─O─C stretching), 1640 cm^−1^ (amide I), and 1540 cm^−1^ (amide II), consistent with protein‐specific amide bonds. Compared to pure ZIF‐67, the enhanced intensity of these peaks in the composite confirms successful enzyme encapsulation. Thermogravimetric analysis (TGA, Figure S15b) showed a three‐stage decomposition process for both materials. In the first stage (near 200°C), ZIF‐67 lost ∼10 wt.% due to bound water release, while Rh‐L6‐FDH@ZIF‐67 lost ∼5 wt.%, indicating initial composite decomposition. The second stage (200°C–600°C) involved rapid weight loss (∼30 wt.% for ZIF‐67, ∼25 wt.% for the composite), attributed to further dehydration and protein degradation [37]. Above 600°C, weight stabilized as decomposition completed. Brunauer–Emmett–Teller (BET) nitrogen sorption isotherms (Figure S15c,d) confirmed mesoporosity, with pore sizes ranging from 1 to 20 nm. Rh‐L6‐FDH@ZIF‐67 exhibited a type IV isotherm with an H4 hysteresis loop and an average pore size of 9.86 nm, which supports efficient diffusion of substrates such as NAD^+^ to the encapsulated catalytic sites [38].

**FIGURE 4:**
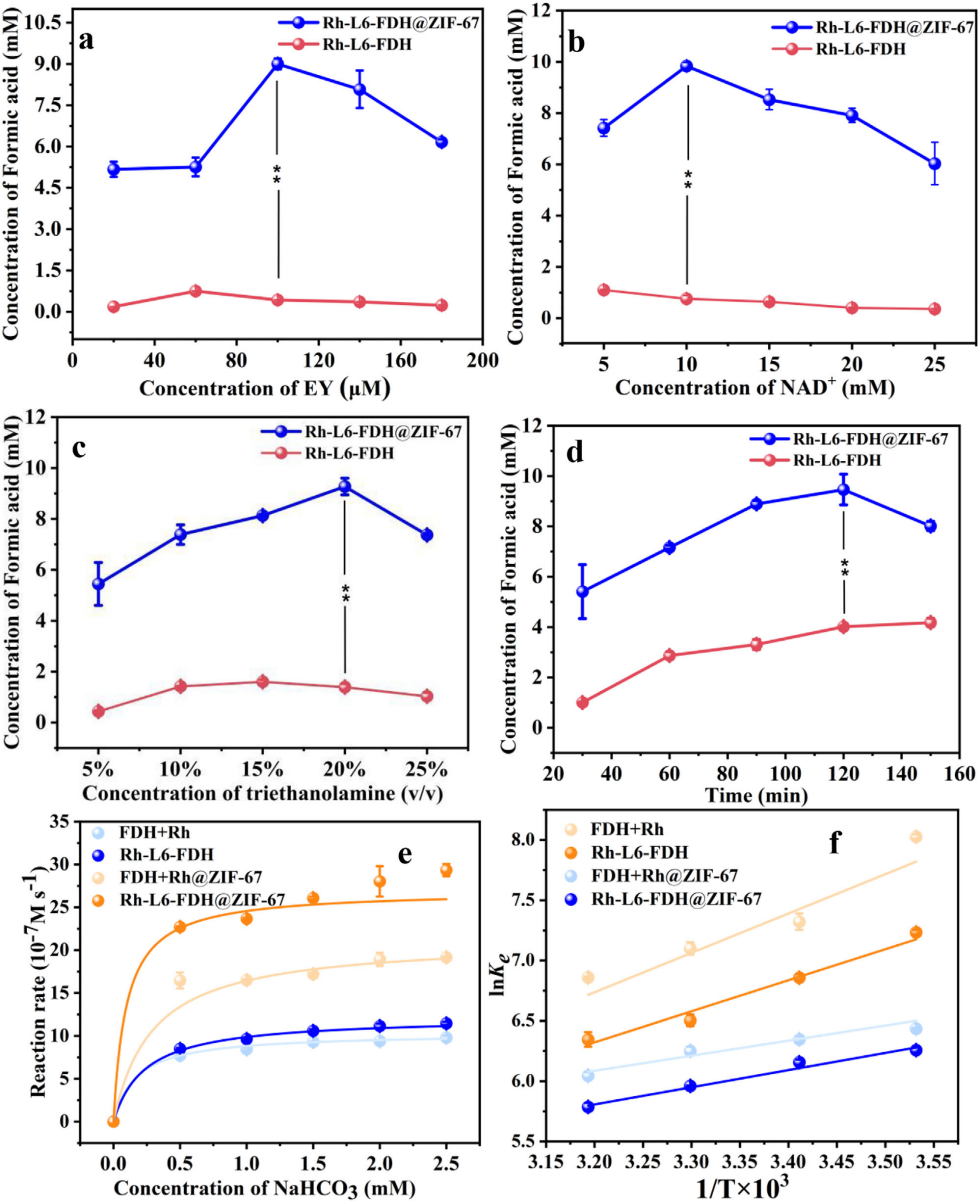
Catalytic properties of bifunctional Rh‐L6‐FDH and Rh‐L6‐FDH@ZIF‐67. (a) Effect of different EY concentrations on photocatalysis. Error bars: mean ± SD (*n* = 3). Statistical significance: **p < 0.01 (t‐test). (b) Effect of different NAD^+^ concentrations on photocatalysis. Error bars: mean ± SD (*n* = 3). Statistical significance: **p < 0.01 (t‐test). (c) Effect of different TEOA concentrations on photocatalysis. Error bars: mean ± SD (*n* = 3). Statistical significance: **p < 0.01 (t‐test). (d) Effect of different light durations on photocatalysis. Error bars: mean ± SD (*n* = 3). Statistical significance: **p < 0.01 (t‐test). (e) The reaction rates of free FDH + Rh‐L6, FDH + Rh‐L6@ZIF‐67, Rh‐L6‐FDH, and Rh‐L6‐FDH@ZIF‐67 with different concentrations of the NaHCO_3_ substrate. (f) Linear fits of *lnK_e_* and *1/T* for free FDH + Rh‐L6, FDH + Rh‐L6@ZIF‐67, Rh‐L6‐FDH, and Rh‐L6‐FDH@ZIF‐67.

A stable microenvironment is critical for maintaining enzymatic catalytic efficiency and is highly dependent on the physicochemical conditions of the reaction system. As the photocatalyst, EY concentration directly influences the rate of electron generation, which governs NADH regeneration. The formic acid yield was evaluated under varying photosensitizer Eosin Y (EY) concentrations (Figure 4a). Rh‐L6‐FDH@ZIF‐67 achieved a maximum yield of 6.19 mM (61.9% conversion) at 60 µM EY. Lower EY concentrations led to slow electron photogeneration, failing to support sufficient NADH regeneration. Conversely, excessive EY induced severe self‐quenching and loss of photogenerated electrons [39]. Due to the intrinsic toxicity of EY, the free Rh‐L6‐FDH system showed relatively low formic acid production.

The coenzyme NAD^+^ is reduced to NADH through H^+^ acquisition mediated by the rhodium complex, which subsequently drives the enzymatic conversion of CO_2_ into formic acid. Rh‐L6‐FDH@ZIF‐67 achieved a maximum formic acid yield of 9.84 ± 0.141 mM (98.4% conversion) at 10 mM NAD^+^, which is 12.98‐fold higher than that of free Rh‐L6‐FDH under identical conditions (Figure 4b). Insufficient NAD^+^ limits both NADH regeneration and proton supply, thereby restricting formic acid production, whereas excessive NAD^+^ may accumulate and drive the reverse reaction—reconverting formic acid to CO_2_. Furthermore, the free enzyme system may create locally elevated NAD^+^ concentrations around the ArM, further suppressing formic acid formation [40].

In the photocatalytic system, triethanolamine (TEOA) serves as a sacrificial electron donor. It accepts H^+^ from the photosensitizer EY, allowing electrons from EY to participate in NAD^+^ reduction to NADH. TEOA exhibits notable advantages, including exceptional electron‐donating capability, favorable aqueous solubility, relatively low toxicity, and the ability to inhibit photodegradation of photosensitizers. However, it is also accompanied by limitations such as the tendency to induce undesirable side reactions, potential alteration of the pH environment, and concentration‐dependent quenching effects that may reduce photocatalytic efficiency. The formic acid yield of Rh‐L6‐FDH@ZIF‐67 first increased and then decreased with rising TEOA concentration (Figure 4c), reaching a maximum of 9.27 mM (92.7% conversion) at 20% (v/v) TEOA. Free Rh‐L6‐FDH exhibited a similar trend, with an optimal concentration at 10% (v/v). The formic acid yield also displayed an initial increase followed by a decline over time (Figure 4d). The rhodium complex facilitates directional electron transfer to NAD^+^, thereby enhancing NADH regeneration. The highest yield for Rh‐L6‐FDH@ZIF‐67 was 9.46 mM (94.6% conversion) at 150 min, a trend consistent with that observed for the free enzyme.

The catalytic kinetics of formic acid production by both free and ZIF‐67‐immobilized bifunctional Rh‐L6‐FDH conform to the Michaelis–Menten model (Figure 4e). Under optimized conditions, the *K_m_* values for free FDH + Rh‐L6, FDH + Rh‐L6@ZIF‐67, free Rh‐L6‐FDH, and Rh‐L6‐FDH@ZIF‐67 were determined as 0.170, 0.292, 0.236, and 0.098 mM, respectively (Table S2). The lower *K_m_* of Rh‐L6‐FDH@ZIF‐67 likely arises from the enrichment of substrate (HCO_3_^−^/CO_2_) within the hydrophobic pores of ZIF‐67, which enhances local substrate concentration near the active sites. Meanwhile, the increase *K_m_* of the free FDH + Rh‐L6@ZIF‐67 system suggests that mass transfer limitations become more pronounced when the enzyme is confined within the framework. The corresponding *k_cat_* values—indicating the turnover number of CO_2_ per enzyme per second—were 0.413, 0.839, 0.488, and 1.066 s^−1^, respectively. These results reveal that free Rh‐L6‐FDH exhibits higher catalytic efficiency than the unconjugated FDH + Rh‐L6 system. Moreover, encapsulation within ZIF‐67 significantly improved structural stability against thermal, pH, and solvent‐induced denaturation, thereby maintaining enzyme activity under harsh conditions and further boosting catalytic performance. Among all systems, Rh‐L6‐FDH@ZIF‐67 showed the highest *V_max_*, underscoring the protective role of ZIF‐67 in preventing enzyme inactivation due to environmental acidification and thus sustaining a stable formic acid production rate. In addition, the cobalt ions in ZIF‐67 offer photoactive sites that promote the generation of photogenerated electron–hole pairs under light irradiation. This led to faster electron transfer in Rh‐L6‐FDH@ZIF‐67 than that in the free system, further accelerating formic acid formation. The overall catalytic efficiency, represented by *k_cat_/K_m_*, was markedly higher for Rh‐L6‐FDH@ZIF‐67 than for the other three systems. This enhancement can be attributed to the increased substrate accessibility to active sites and the effective preservation of enzymatic activity afforded by the immobilized structure [41].

The catalytic thermodynamic properties of free FDH + Rh, FDH + Rh@ZIF‐67, Rh‐L6‐FDH, and Rh‐L6‐FDH@ZIF‐67 for formic acid synthesis were evaluated (Figure 4f). The process followed the Arrhenius equation, with linear fits between ln*K_e_* and 1/*T* yielding *R^2^* values of 0.8683, 0.9819, 0.9709, and 0.9279, respectively. The corresponding activation energies were calculated as −21.749, −10.401, −21.397, and −11.848 J·mol^−1^. The lower activation energy of Rh‐L6‐FDH@ZIF‐67 compared to free Rh‐L6‐FDH indicates a reduced energy barrier and enhanced catalytic efficiency, likely due to the porous structure of ZIF‐67 providing greater access to active sites [42]. Thermodynamic parameters (*ΔH*, *ΔS*, *ΔG*) of all systems showed negative *ΔH*, positive *ΔS*, and negative *ΔG*, confirming that the reactions are exothermic and spontaneous (Table S3). The smallest *ΔS* for free FDH + Rh suggests the least disorder during catalysis. Rh‐L6‐FDH@ZIF‐67 exhibited a smaller absolute *ΔG* value than Rh‐L6‐FDH, reflecting a stronger driving force for the reaction. Although encapsulation within ZIF‐67 may restrict substrate diffusion, it also resulted in a smaller absolute *ΔH*, indicating milder heat release and improved system stability. Overall, the thermodynamic analysis confirms that ZIF‐67 immobilization enhances the stability of Rh‐L6‐FDH.

The Rh‐L6‐FDH@ZIF‐67 exhibits enhanced pH and thermal stability (Figure S16), and maintains good enzyme activity in different organic solvents and the catalytic efficiency of Rh‐L6‐FDH@ZIF‐67 remained above 41.3% after 5 consecutive recovery steps (Figure S17). Structural damage from mechanical stress combined with the accumulation of reaction products or by‐products within the carrier pores, can contribute to a progressive decline in enzymatic activity during repeated cycles. Meanwhile, the preparation process of ZIF‐67 faces scalability limitations, with narrow optimal synthesis conditions that make it difficult to achieve quality control in large‐scale production. The transition from laboratory achievements to large‐scale industrial applications remains challenging [43]. This finding indicated that Rh‐L6‐FDH@ZIF‐67 provides a stable protective microenvironment in the face of harsh catalytic conditions, thus maintaining high reusability.

Moreover, by encapsulating the ArM within a ZIF‐67 matrix, the catalyst was effectively shielded from microenvironmental perturbations, thereby enhancing its operational stability. In this synergistic multi‐component system, the photosensitizer EY absorbs light energy to generate photoexcited electrons, while the sacrificial agent TEOA promotes the regeneration of the photosensitizer. The Rh‐L6 complex subsequently accepts these electrons along with protons (H^+^), enabling the selective reduction of NAD^+^ to NADH. Incorporated within an ArM, the Rh‐L6 complex efficiently catalyzes the conversion of NAD^+^ to NADH, thereby supplying sufficient coenzyme for CO_2_ reduction facilitated by FDH and significantly enhancing the overall catalytic efficiency.

## 3 Conclusion

In this study, we report the first development of a novel rhodium‐based artificial metalloenzyme designed for efficient light‐driven CO_2_ reduction to formic acid coupled with NADH regeneration. This integrated system significantly reduces dependence on expensive exogenous cofactors while substantially boosting product yield. By encapsulating the artificial metalloenzyme within a ZIF‐67, we effectively shielded the catalyst from microenvironmental perturbations, thereby enhancing its operational stability. The exceptional performance of this nanoreactor—achieving a remarkable CO_2_‐to‐formic acid conversion efficiency of 98.4%—is driven by a synergistic interplay of three key factors: (1) the proximity effect between the metal and enzymatic catalytic centers, which facilitates rapid electron transfer; (2) the kinetic coordination between NADH regeneration and consumption rates; and (3) the confinement effect provided by the stable nano‐structured microenvironment. These findings underscore the immense potential of hybrid bio‐hybrid systems in advancing sustainable catalysis.

## 4 Methods

### 4.1 Preparation of the Artificial Metalloenzyme Rh‐L6‐FDH

A pure FDH enzyme solution (final concentration: 25 µM) and the rhodium complexes Rh‐L4, Rh‐L5, and Rh‐L6 (final concentration: 25 µM) were added to phosphate buffer (50 mM, pH 7.2) and incubated at 4°C for 1 h. Following incubation, unbound metals were removed using a Sephadex G‐25 desalting column. The resulting artificial metalloenzymes were designated Rh‐L4‐FDH, Rh‐L5‐FDH, and Rh‐L6‐FDH, respectively.

To extend the linker length between the rhodium complexes and the enzyme FDH, the protein cross‐linkers Succinimidyl 4‐(N‐maleimidomethyl)cyclohexane‐1‐carboxylate (SMCC) and Disuccinimidyl Suberate (DSS) were employed, enabling the coupling of two compounds containing sulfhydryl and amino groups or two amino groups, respectively. Three additional rhodium complexes, Rh‐L1, Rh‐L2, and Rh‐L3, were synthesized, all bearing amino groups, allowing covalent attachment to the enzyme via SMCC‐mediated coupling to sulfhydryl groups (the resulting artificial metalloenzymes were designated Rh‐L7‐FDH, Rh‐L8‐FDH, and Rh‐L9‐FDH, respectively) or DSS‐mediated coupling through amino groups (designated Rh‐L10‐FDH, Rh‐L11‐FDH, and Rh‐L12‐FDH, respectively). The total system volume was maintained at 5.0 mL; pure FDH enzyme solution (final concentration: 25 µM) and the protein cross‐linker (final concentration: 25 µM) were added to phosphate buffer (50 mM, pH 7.2) and incubated at 4°C for 1 h, after which the rhodium complexes (final concentration: 25 µM) were added and incubation continued under the same conditions for an additional hour.

The optimal synthesis conditions for the artificial metalloenzyme Rh‐L6‐FDH involve a molar ratio of FDH to Rh‐L6 of 1:3, with all other conditions held constant. The unbound metal was then removed by using a SephadexG‐25 desalting column.

### 4.2 Preparation of Rh‐L6‐FDH@ZIF‐67

A solution of 2‐methylimidazole (100 mM, 0.5 mL) was mixed with the Rh‐L6‐FDH solution in a 20 mL glass vessel and stirred at 25°C and 200 rpm for 5 min. Subsequently, cobalt chloride solution (CoCl_2_, 50 mM, 1.0 mL) was added, and ultrapure water was used to adjust the total volume to 5.0 mL. The stirring speed was then increased to 500 rpm and maintained for 30 min. Upon completion, the mixture was centrifuged at 6000 rpm and 4°C for 10 min to remove the supernatant. The resulting pale purple precipitate was collected and washed three times with ultrapure water.

### 4.3 Photocatalytic Reaction

The reaction was conducted in a 20 mL glass reactor under illumination from a 350 W full‐wavelength xenon lamp with a color temperature of 5600 K and a cutoff filter (>420 nm). The reaction mixture consisted of 50 mM PBS buffer (pH 7.2), 10 mM NaHCO_3_, 60 µM EY, 4 mM NAD+, and 15% (v/v) TEOA. The temperature was maintained at 40°C via a circulating water system.

The formate was quantified via an Agilent 1290 HPLC system (Figure S18) equipped with an Aminex HPX‐87H column (300 × 7.88 mm^2^, 5 µm). The column temperature was 40°C, the mobile phase was 4.0 mM sulfuric acid, the flow rate was 0.6 mL/min, the injection volume was 10 µL, and the UV detector wavelength was set at 210 nm.

### 4.4 Molecular Simulation of Artificial Metalenzyme

Molecular dynamics (MD) simulations were performed using GROMACS 2021. All systems were simulated under periodic boundary conditions at 300 K and 1.0 bar, with a minimum distance of 1.0 nm between the protein and box edges. The topology of the Rh‐L6 complex was converted to GROMACS format using pdb2gmx, employing the AMBER ff14SB force field for the protein. Non‐standard residues were processed with AmberTools and the GAFF force field to generate ligand topologies, and the TIP3P water model was used.

For density functional theory (DFT) calculations, a representative configuration was extracted from docking results. For Rh‑L6‑FDH, residues surrounding the [(Cp*)Rh(bpy)Cl]+ center were retained. Geometry optimizations were carried out at the M06‑2X/6‑31G* level with the LanL2DZ effective core potential (ECP) for Rh. Frequency calculations confirmed all optimized structures as true minima (zero imaginary frequencies). Single‐point energies were then computed at the M06‑2X/def2‑TZVP level with the SDD ECP for Rh, incorporating solvation via the PCM model.

For NAD^+^ reduction modeling, a truncated model—1‑methylnicotinamide (ribose replaced by a methyl group)—was used. Geometry optimization employed B3LYP(D3BJ)/6‐31G* with LanL2DZ ECP for Rh, followed by frequency analysis (zero imaginary frequencies). Single‑point energies were calculated at the B3LYP(D3BJ)/def2‐TZVP level with LanL2DZ ECP for Rh, again using PCM solvation.

### 4.5 Statistical Analysis

Statistical analyses were performed using Origin2019. Data are expressed as mean ± SD from three independent experiments (*n* = 3). Unless otherwise stated, no data transformation, normalization, or outlier exclusion was applied. For two‐group comparisons, an unpaired two‐tailed Student's t‐test was used where appropriate. Kinetic parameters were determined by nonlinear regression. All tests were two‐sided, with *p* ≤ 0.05 considered significant and *p* ≤ 0.01 considered highly significant.

## Author Contributions

**Mengxue Kang**: methodology, investigation, writing – original draft, formal analysis. **Yan Chu**: methodology, investigation, writing – original draft, data curation. **Zhichun Xu**: investigation, data curation. **Jiao Feng**: funding acquisition, resources. **Ganlu Li**: project administration, resources, supervision. **Kequan Chen**: funding acquisition, project administration, supervision. **Hui Li**: writing – review and editing, funding acquisition, conceptualization, project administration, supervision, resources.

## Conflicts of Interest

The authors declare no conflicts of interest.

## Supporting information




**Supporting File**: advs78027‐sup‐0001‐SuppMat.docx

## Data Availability

The data that support the findings of this study are available from the corresponding author upon reasonable request.
