# Supplementary material for: Engineering an Artificial Metalloenzyme for Efficient Photo‐Enzymatic CO2 Conversion
Source: Adv Sci (Weinh). 2026 Sep 27:e78027. Online ahead of print. doi: 10.1002/advs.78027 (PMC13616261; doi:10.1002/advs.78027)
Supplement: Supplementary file 1 [file ADVS-9999-e78027-s001.docx]

Supplementary Materials for

**Engineering an Artificial Metalloenzyme for Efficient Photo-Enzymatic CO₂ Conversion**

Mengxue Kang^1^, Yan Chu^1^, Zhichun Xu, Jiao Feng, Ganlu Li, Kequan Chen^*^, Hui Li^*^

College of Biotechnology and Pharmaceutical Engineering, State Key Laboratory of Materials–Oriented Chemical Engineering, Nanjing Tech University, Nanjing 211816, China

^1^ These authors contributed equally to this work.

^*^ Corresponding author.

E-mail address: lihui11@njtech.edu.cn (H. Li), kqchen@njtech.edu.cn (K. Chen)

**Table of Contents**

[Supplementary Methods	2](#_Toc18067)

[Supplementary Method 1 – Preparation of formate dehydrogenase (FDH)	2](#_Toc8831)

[Supplementary Method 2 – Preparation of rhodium complexes	3](#_Toc737)

[Supplementary Method 3 - Determination of NADH concentration	10](#_Toc14200)

[Supplementary Method 4 - Desalination of artificial metalloenzymes	10](#_Toc16881)

[Supplementary Method 5 - SDS-PAGE of Rh-L6-FDH	10](#_Toc28030)

[Supplementary Method 6 - Optical properties of artificial metalloenzymes	11](#_Toc32477)

[Supplementary Method 7 - Photoelectrochemical performance of artificial metalloenzymes	11](#_Toc28637)

[Supplementary Method 8 - Synthesis optimization of Rh-L6-FDH@ZIF-67	12](#_Toc18934)

[Supplementary Method 9 - Stability experiments of Rh-L6-FDH@ZIF-67	12](#_Toc7948)

[Supplementary Method 10 - Enzymatic properties of Rh-L6-FDH@ZIF-67	13](#_Toc30108)

[Supplementary Method 11 - Characterization	13](#_Toc12451)

[Supplementary Figures	14](#_Toc17107)

[Supplementary Tables	23](#_Toc1061)

# **Supplementary Methods**

## Supplementary **Method** 1 – Preparation of formate dehydrogenase (FDH)

Glycerol bacteria stored in FDH (Gelatoporia subvermispora) were spread on LB solid medium containing kanamycin, placed in an incubator at 37 ℃, and cultured overnight for 12 hours to obtain a single colony. A single colony was inoculated it into 5.0 mL LB medium containing 50 mg/L kanamycin, placed in a shaker at 37 ℃ for 12 hours, transferred into 100 mL LB liquid medium at 37 ℃ and 200 rpm, and continued to cultivate it for 2-3 hours. When its OD_600_ reached 0.6-0.8, the protein inducer isopropyl thiogalactoside (IPTG) at a final concentration of 0.1 mM was added at 18 ℃ and 200 rpm for 12 to 14 hours, and the above culture was centrifuged to obtain cells. 50 mM PBS buffer with pH 7.2 is used to resuspend the cells, and the crushing needs to be carried out at low temperatures. The cell granules were thawed on ice and resuspended in lysis buffer (50 mM PBS buffer, pH 7.2). The cells were then disrupted using a SCIENTZ-IID ultrasonic cell breaker (Ningbo, China) and cooled on ice. Cell lysate was centrifuged at 6000 rpm for 30 min at 4 °C to remove cell debris. The supernatant was filtered through a 0.22 μm PVDF filter and loaded into a Ni-NTA chromatography column (HitrapQFF) equilibrated with lysis buffer. After loading the filtered lysate, the miscellaneous proteins were washed with washing buffer (50 mM PBS buffer containing 50 mM imidazole, pH 7.0), and then eluted buffer (50 mM PBS buffer containing 300 mM imidazole, pH 7.0). Finally, the eluate was ultrafiltered with a Millipore ultrafiltration membrane to remove imidazole from the wash buffer, and the FDH-containing fractions were pooled in Tris-HCl buffer (pH 7.0) at 4 °C for later use.

## Supplementary **Method** 2 – Preparation of rhodium complexes

2.1 Preparation of ligand L4

A three-necked flask was filled with 30 mL of anhydrous CHCl_3_, and 85.6 mg (0.5 mmol) of 4-amino-2, 2’-bipyridine and 123 mg (0.6 mmol; 1.2 eq) of bromoacetyl bromide were added at room temperature under nitrogen. Filter immediately when white suspension appears. The filtrate was stirred for 30 min and then acidified with 10 mL of 0.1 M HCl. The organic layer was extracted twice with 0.1 M HCl. The combined aqueous phases were basified to pH 8.0 with 0.1 wt% sodium bicarbonate. The aqueous phase was extracted three times with CHCl_3_ and dried over anhydrous sodium sulfate. Concentration in vacuo gave a white solid.

MS(ESI)：m/z=306.0504[M]^+^

**Figure S1** Mass spectrometry of ligand L4

2.2 Preparation of ligand L5

A three-necked flask was filled with 30 mL of anhydrous CHCl_3_, 94 mg (0.5 mmol) of 5, 5’-diamino-2, 2’-bipyridine and 123 mg (0.6 mmol; 1.2 eq) of bromoacetyl bromide were added at room temperature under nitrogen. Filter immediately when white suspension appears. The filtrate was stirred for 30 min and then acidified with 10 mL of 0.1 M HCl. The organic layer was extracted twice with 0.1 M HCl. The combined aqueous phases were basified with 0.1 wt% sodium bicarbonate to pH 8.0. The aqueous phase was extracted three times with CHCl_3_ and dried over anhydrous sodium sulfate. Concentration in vacuo gave ligand L5 as a white solid.

MS(ESI)：m/z=326.0013[M]^+^

**Figure S2** Mass spectrometry of ligand L5

2.3 Preparation of ligand L6

Under a nitrogen atmosphere, 195 mg (1.0 mmol) of 1, 10-phenanthroline-5-amine was added to a two-necked flask containing 30 mL of anhydrous CHCl_3_. 242 mg (1.2 mmol; 1.2 eq) of bromoacetyl bromide was slowly added. The reaction mixture was heated at reflux overnight and the resulting suspension was filtered. The solid was obtained by rotary evaporation under vacuum and then recrystallized from methanol to obtain a yellow solid.

MS(ESI): m/z=316.6703[M]^+^

**Figure S3** Mass spectrometry of ligand L6

2.4 Preparation of ligand Rh-L1

Under a nitrogen atmosphere, pentamethylcyclopentadienyl rhodium (III) chloride dimer (100 mg, 0.162 mmol, 1.0 eq) was suspended in methanol (2.0 mL). A methanol solution (1.0 mL) of 4-amino-2, 2’-bipyridine (56 mg, 0.324 mmol, 2.0 eq) was added to the suspension. The mixture immediately turned into orange transparent solution, indicating the formation of a rhodium-bipyridine complex. Concentration in vacuum gave a yellow powder Rh-L1.

MS(ESI)：m/z=444.7358[M]^+^

**Figure S4** Mass spectrometry of ligand Rh-L1

2.5 Preparation of ligand Rh-L2

Under a nitrogen atmosphere, pentamethylcyclopentadienyl rhodium (III) chloride dimer (100 mg, 0.162 mmol, 1.0 eq) was suspended in methanol (2.0 mL). A methanol solution (1.0 mL) of 5, 5’-diamino-2, 2’-bipyridine (61 mg, 0.324 mmol, 2.0 eq) was added to the suspension. The mixture immediately turned into orange transparent solution, indicating the formation of a rhodium-bipyridine complex. Concentration in vacuum gave a yellow powder Rh-L2.

MS(ESI)：m/z=459.1754 [M]^+^

**Figure S5** Mass spectrometry of ligand Rh-L2

2.6 Preparation of ligand Rh-L3

Under a nitrogen atmosphere, pentamethylcyclopentadienyl rhodium (III) chloride dimer (100 mg, 0.162 mmol, 1.0 eq) was suspended in methanol (2.0 mL). A methanol solution (1.0 mL) of 1, 10-phenanthroline-5-amine (64 mg, 0.324 mmol, 2.0 eq) was added to the suspension. The mixture immediately turned into orange transparent solution, indicating the formation of a rhodium-phenanthroline complex. Concentration in vacuum gave a yellow powder of Rh-L3.

MS(ESI)：m/z=469.3324[M]^+^

**Figure S6** Mass spectrometry of ligand Rh-L3

2.7 Preparation of ligand Rh-L4

Under a nitrogen atmosphere, pentamethylcyclopentadienyl rhodium (III) chloride dimer (100 mg, 0.162 mmol, 1.0 eq) was suspended in methanol (2.0 mL). A methanol solution (1.0 mL) of ligand L4 (100 mg, 0.324 mmol, 2.0 eq) was added to the suspension. The mixture immediately turned into orange transparent solution, indicating the formation of a rhodium-bipyridine complex. Concentration in vacuum gave a yellow powder of Rh-L4.

MS(ESI)：m/z=576.7819[M]^+^

**Figure S7** Mass spectrometry of ligand Rh-L4

2.8 Preparation of ligand Rh-L5

Under a nitrogen atmosphere, pentamethylcyclopentadienyl rhodium (III) chloride dimer (100 mg, 0.162 mmol, 1.0 eq) was suspended in methanol (2.0 mL). A methanol solution (1.0 mL) of ligand L5 (105 mg, 0.324 mmol, 2.0 eq) was added to the suspension. The mixture immediately turned into orange transparent solution, indicating the formation of a rhodium-bipyridine complex. Concentration in vacuum gave a yellow powder Rh-L5.

MS(ESI)：m/z=595.4442[M]^+^

**Figure S8** Mass spectrometry of ligand Rh-L5

2.9 Preparation of ligand Rh-L6

Under a nitrogen atmosphere, pentamethylcyclopentadienyl rhodium (III) chloride dimer (100 mg, 0.162 mmol, 1.0 eq) was suspended in methanol (2.0 mL). A methanol solution (1.0 mL) of ligand L6 (102 mg, 0.324 mmol, 2.0 eq) was added to the suspension. The mixture immediately turned into orange transparent solution, indicating the formation of a rhodium-bipyridine complex. Concentration in vacuum gave a yellow powder Rh-L6.

MS(ESI)：m/z=587.9505 [M]^+^

**Figure S9** Mass spectrometry of ligand Rh-L6

## **Supplementary Method 3** - Determination of NADH concentration

NADH standard solutions with different concentrations (0.1-0.5mM) were prepared and placed them in a 96-well plate. A microplate reader was used to measure its absorbance at 340 nm. The NADH contents of the samples were then measured according to the above method and calculated into the standard curve (y = 0.2869x-0.0173, *R²* = 0.9958).

## Supplementary **Method** 4 - Desalination of artificial metalloenzymes

The insoluble impurities in Rh-L6-FDH solution were removed by centrifugation at 12000 rpm for 5 min, and then the desalting column was equilibrated with PBS buffer. After the sample was immersed in the desalting column, the PBS buffer was poured into the column for elution, and a centrifuge tube was placed at the bottom of the desalting column to collect the eluent, which was then stored at 4 ℃.

## Supplementary **Method 5** - SDS-PAGE of Rh-L6-FDH

(1) Sample preparation and spiking: 20 μL of protein sample was took and 4 μL of 6×sampling buffer was added, mixed well and boiled it in boiling water for 10 min, Tris-Gly electrophoresis buffer was poured into the electrophoresis tank, and 5 μL of Protein Marker and 20 μL of denatured protein samples were added sequentially in the sample tank.

(2) Electrophoresis: The electrophoresis voltage was set to 150 V to run for 15 min, and after the protein samples entered the separation gel, the voltage was set to 220 V to run for 40 min, and the electrophoresis was stopped when the blue dye band migrated to a position about 1.0 cm from the lower end.

(3) Staining and decolorization: The electrophoresis gel was removed and placed in the staining box. The staining solution was added for 30 min and recycled, and then the decolorizing solution was added, replaced it every 2 h. The process was repeated for 3 times and then decolorized overnight until the background was clear.

## Supplementary **Method 6** - Optical properties of artificial metalloenzymes

Shimadzu UV-3600i Plus UV-visible spectrophotometer was used for the test. 5 mg of sample powder was accurately weighed and evenly spread on the surface of a BaSO_4_ standard white plate, and the test samples with consistent thickness were prepared using a special tablet pressing device. The instrument parameters were set to a wavelength range of 200-800 nm, a scanning speed of 1.0 nm∙s^-1^, and at least three repetitions for each sample.

The optical band gap of the material is calculated by Tauc plot method using the UV absorption spectrum energy, most of the valence band electrons and conduction band electrons of the material are distributed in the vicinity of the forbidden band, so when the photon energy is close to the width of the forbidden band, a large number of electrons can jump by absorbing the photon energy, and at this time the absorption coefficient will be increased with the increase in the number of photons, and there is a relationship between its optical band gap and absorption coefficient as follows:

$$\text{ }\text{ (αhν)\textasciicircum1/n=B(hν-}\text{E}_{\text{g}}\text{) }\text{(1)}$$

Where α is the absorption coefficient, *hν* is the photon energy, *h* is Planck's constant, *ν* is the incident photon frequency, *B* is the proportionality constant, and *E_g_* is the forbidden bandwidth of the semiconductor material.

## Supplementary **Method 7** - Photoelectrochemical performance of artificial metalloenzymes

Samples were tested for photoluminescence spectroscopy (PL) using the Edinburgh FLS1000 at -196 °C with an excitation wavelength of 375 nm. PL was performed along with exciton lifetime testing. The test method was as follows: 4 mg of sample, 0.375 mL of ethanol, 0.125 mL of water, and 50 μL of 5% (v/v) Nafion solution were mixed and sonicated for 1.0 h. The mixture was dripped onto the surface of a glassy carbon electrode and dried at room temperature before use. The test system was a standard three-electrode system, the working electrode was a glassy carbon electrode loaded with sample, the counter electrode was a platinum sheet electrode, the reference electrode was an Ag/AgCl electrode, the electrolyte was a 0.5 M Na_2_SO_4_ solution, and the electrochemical workstation was a Princeton ParSTAT MC.

## Supplementary **Method 8** - Synthesis optimization of Rh-L6-FDH@ZIF-67

Differences in the synthesis conditions of the enzyme immobilization will affect the effect of the enzyme immobilization. The wrapping ability of ZIF-67 is a decisive factor for the enzyme activity. The too loose structure is poor protection for the enzyme, which affects the overall enzyme's formic acid conversion. The effect of the 2-methyl imidazole (2-MM) concentration, Co^2+^ concentration, stirring speed and synthesis time on the formic acid conversion rate were studied (Figure S12). The highest value of 80.43% formic acid conversion was achieved when the 2-MM concentration was 100 mM, Co^2+^ concentration was 60 mM, stirring speed was 100 rpm and synthesis time was 40 min. The structure of Rh-L6-FDH@ZIF-67 synthesized under this condition was the most stable, and it was not easy to have the problems of material collapse and too small pores, and the enzyme could be completely wrapped in ZIF-67, which was not easy to cause inactivation.

## Supplementary **Method 9** - Stability experiments of Rh-L6-FDH@ZIF-67

A stable microenvironment is a decisive factor for the catalytic efficiency of the enzyme. pH affects the dissociation of polar groups on the side chains of enzyme molecules, changing their charged state and thus the structure of the enzyme activity centers, as well as affecting the degree of dissociation of the substrate molecules and coenzymes. Figure S16a-b shows the effect of pH on the Rh-L6-FDH and Rh-L6-FDH@ZIF-67 enzyme stability. Enzyme immobilization improves the stability of the Rh-L6-FDH to some extent, and by comparison, the enzyme is more suitable for reaction in alkaline environment. Temperature affects the binding rate of enzyme to substrate. Figure S16c-d shows the effect of temperature on enzyme stability of Rh-L6-FDH and Rh-L6-FDH@ZIF-67. The enzyme stability is better in 40°C, indicating that high temperature causes the resultant cleavage of ZIF-67, with a large amount of enzyme spillover and inactivation by loss of material protection.

## Supplementary **Method** 10 - Enzymatic properties of Rh-L6-FDH@ZIF-67

FDH, Rh-L6-FDH, and Rh-L6-FDH@ZIF-67 were incubated in organic reagents methanol, N, N-dimethylformamide (DMF), dimethylsulfoxide (DMSO), and isopropanol for 30 min in PBS buffer (pH 6.0) with 50 mM buffer (pH 6.0) using NaHCO_3_ as the substrate under optimal conditions for catalysis, defining the enzyme activity as 100% when no organic reagents were added. Figure S8a shows that the enzyme activities of FDH and Rh-L6-FDH in methanol solvents are stable with essentially no decrease, whereas in isopropanol the enzyme activity is only about 20%, causing irreversible inactivation of the enzyme structure.

The catalytic experiments were repeated several times under optimal catalytic conditions, and at the end of each reaction, the catalyst was centrifugally washed three times with ultrapure water and reintroduced into the reaction system. All reactions were carried out in a buffer solution of 50 mM PBS (pH 6.0). Figure S8b performed the reuse of Rh-L6-FDH@ZIF-67. Studies showed that after 2 iterations, the enzyme activity of Rh-L6-FDH@ZIF-67 was 81.31%. After 5 iterations, the relative enzyme activity of Rh-L6-FDH@ZIF-67 was still 41.13%.

## Supplementary **Method** 11 - Characterization

Gel permeation chromatography (GPC) was performed using a Waters 2695 HPLC system equipped with a TSKgel G2000 SWxL column. UV-Vis spectra were recorded using a UV-3600 spectrophotometer. Circular dichroism (CD) measurements were carried out with a Jasco J-815 spectropolarimeter. Fourier-transform infrared (FTIR) analysis was conducted using a Spectrum 100 spectrometer. Scanning electron microscopy (SEM) and transmission electron microscopy (TEM) were performed with S4800 and JEM-1400UHR microscopes coupled with Energy Dispersive Spectrometer (EDS), respectively. X-ray Photoelectron Spectroscopy (XPS) analysis was done with a K-Alpha XPS spectrometer. Thermogravimetric analysis (TGA) was performed on a chirascan V100 thermogravimetric analyzer under nitrogen from 25 ℃ to 800 ℃ at 10 ℃/min. BET analysis was conducted with a BELSORP-miniX instrument.

# Supplementary Figures

**c**

**b**

**d**

**a**

**Figure S10 a**. Effect of different rhodium complexes on enzyme activity and NADH regeneration capacity. **b.** Formate production by artificial metalloenzymes with different rhodium complexes. **c.** Sodium dodecyl sulfate polyacrylamide gel electrophoresis (SDS-PAGE) of free Rh-L6-FDH and free FDH. **d.** Electrospray ionization-mass spectrometry (ESI-MS) of Rh-L6-FDH.

**b**

**a**

**d**

**c**

**e**

**Figure S11 a.** Changes in Radius of gyration (Rg) of Rh-L6-FDH over time. **b.** Root-mean-square fluctuation (RMSF) values for each amino acid residue of Rh-L6-FDH. **c.** Number of internal hydrogen bonds in Rh-L6-FDH over time. **d.** Solvent Accessible Surface Area (SASA) of Rh-L6-FDH over time. **e.** Variation of secondary residue composition of Rh-L6-FDH over time.

**Figure S12 Optimization of synthesis conditions for Rh-L6-FDH@ZIF-67**: **a.** 2-MM concentration, **b.** Co^2+^ concentration, **c.** rotational speed, **d.** synthesis time.

**Figure S13 Characterization of bifunctional Rh-L6-FDH@ZIF-67.** Scanning electron microscopy (SEM) characterization of **a.** ZIF-67 and **c.** Rh-L6-FDH@ZIF-67. Transmission electron microscopy (TEM) characterization of **b.** ZIF-67 and **d.** Rh-L6-FDH@ZIF-67. Energy dispersive spectroscopy (EDS) characterization of **e.** ZIF-67 and **f.** Rh-L6-FDH@ZIF-67.

**Figure S14** **X-ray photoelectron spectroscopy (XPS) characterization of Rh-L6-FDH@ZIF-67: a.** N1s spectrum, **b.** O1s spectrum, **c.** P2p spectrum, **d.** Rh3d spectrum, **e.** XPS full spectrum.

**d**

**c**

**b**

**a**

**Figure S15 a.** Fourier transform infrared spectroscopy (FTIR) characterization of ZIF-67, Rh-L6-FDH@ZIF-67, and Rh-L6-FDH. **b.** Thermogravimetric analysis (TGA) characterization of ZIF-67 and Rh-L6-FDH@ZIF-67. Brunauer–Emmett–Teller (BET) nitrogen sorption isotherms of **c.** ZIF-67 and **d.** Rh-L6-FDH@ZIF-67.

**Figure S16** **a.** Acid-base stability of Rh-L6-FDH@ZIF-67 at pH 4. **b.** Acid-base stability of Rh-L6-FDH@ZIF-67 at pH 9. **c.** Temperature stability of Rh-L6-FDH@ZIF-67 at T=40°C, **d.** Temperature stability of Rh-L6-FDH@ZIF-67 at T=60°C.

**b**

**a**

**Figure S17** **a.** Organic tolerance test of Rh-L6-FDH@ZIF-67. **b.** Cyclic badness experimental test of Rh-L6-FDH@ZIF-67. **c.** SEM characterization of Rh-L6-FDH@ZIF-67 before reaction. **d.** SEM characterization of Rh-L6-FDH@ZIF-67 after 5 cycles of cyclic badness.

**Figure S18** Liquid chromatogram of formic acid

# Supplementary Tables

**Table S1**

Protein secondary structure data of free Rh-L6-FDH and free FDH from CD

| Samples | *a-*helices | *β-*sheets | Random coil |
| --- | --- | --- | --- |
| Free FDH | 8% | 43% | 48% |
| Free Rh-L6-FDH | 12% | 39% | 48% |

**Table S2**

Catalytic kinetic parameters of Free FDH + Rh, Free Rh-L6-FDH, FDH+Rh@ZIF-67and Rh-L6-FDH@ZIF-67

| Samples | *K_m_*  (mM) | *V_max_*  (10^-7^M∙s^-1^) | *k_cat_*  (s^-1^) | *k_cat_/K_m_*  (s^-1^∙mM^-1^) | *R^2^* |
| --- | --- | --- | --- | --- | --- |
| Free FDH+Rh | 0.171 | 10.33 | 0.413 | 2.415 | 0.9758 |
| Free Rh-L6-FDH | 0.236 | 12.20 | 0.488 | 2.068 | 0. 9534 |
| FDH+Rh@ZIF-67 | 0.292 | 21.23 | 0.839 | 2.871 | 0.9464 |
| Rh-L6-FDH@ZIF-67 | 0.098 | 26.96 | 1.066 | 10.92 | 0.8705 |

**Table S3**

Catalytic thermodynamic parameters of Free FDH + Rh, Free Rh-L6-FDH, FDH+Rh@ZIF-67and Rh-L6-FDH@ZIF-67

| T (K) |  | 283.15 | 293.15 | 303.15 | 313.15 |
| --- | --- | --- | --- | --- | --- |
| FDH+Rh | *∆S* (J∙mol^-1^) | 58.4 | 44.8 | 35.1 | 25.3 |
|  | *∆G* (kJ∙mol^-1^) | -18924.4 | -17957.5 | -17978.7 | -17869.1 |
|  | *∆H* (kJ∙mol^-1^) | -2380.8 | -4817.7 | -7337.7 | -9940.8 |
| FDH@ZIF-67+Rh | *∆S (*J∙mol^-1^) | 1832.4 | 1629.5 | 1270.1 | 944.5 |
|  | *∆G* (kJ∙mol^-1^) | -15136.9 | -15508.1 | -15685.1 | -15683.3 |
|  | *∆H* (kJ∙mol^-1^) | -2364.1 | -4800.9 | -7320.9 | -9924.1 |
| Rh-L6-FDH | *∆S* (J∙mol^-1^) | 1769.6 | 1557.0 | 1279.7 | 901.6 |
|  | *∆G* (kJ∙mol^-1^) | -17058.8 | -16665.2 | -16303.1 | -16408.2 |
|  | *∆H* (kJ∙mol^-1^) | -2375.1 | -4811.9 | -7331.9 | -9935.0 |
| Rh-L6-FDH@ZIF-67 | *∆S* (J∙mol^-1^) | 1928.2 | 1617.7 | 1227.8 | 856.8 |
|  | *∆G* (kJ∙mol^-1^) | -14781.4 | -15040.4 | -15026.4 | -15122.2 |
|  | *∆H* (kJ∙mol^-1^) | -2365.5 | -4802.4 | -7322.4 | -9925.5 |
